# Duality in response of intracranial vessels to nitroglycerin revealed in rats by imaging photoplethysmography

**DOI:** 10.1038/s41598-023-39171-w

**Published:** 2023-07-24

**Authors:** Alexey Y. Sokolov, Maxim A. Volynsky, Anastasiia V. Potapenko, Polina M. Iurkova, Valeriy V. Zaytsev, Ervin Nippolainen, Alexei A. Kamshilin

**Affiliations:** 1 Department of Neuropharmacology, Valdman Institute of Pharmacology, Pavlov First Saint Petersburg State Medical University, Saint Petersburg, Russia; 2 Laboratory of Cortico-Visceral Physiology, Pavlov Institute of Physiology of the Russian Academy of Sciences, Saint Petersburg, Russia; 3 School of Physics and Engineering, ITMO University, Saint Petersburg, Russia; 4 Laboratory of Functional Materials and Systems for Photonics, Institute of Automation and Control Processes of Far East Branch of the Russian Academy of Sciences, Vladivostok, Russia; 5 Laboratory of Biochemistry, Medical Genetic Center, Saint Petersburg, Russia; 6 Faculty of General Therapy, Saint Petersburg State Pediatric Medical University, Saint Petersburg, Russia

**Keywords:** Imaging, Image processing, Neuro-vascular interactions, Migraine, Migraine, Pharmacodynamics

## Abstract

Among numerous approaches to the study of migraine, the nitroglycerin (NTG) model occupies a prominent place, but there is relatively insufficient information about how NTG affects intracranial vessels. In this study we aim to assess the effects of NTG on blood-flow parameters in meningeal vessels measured by imaging photoplethysmography (iPPG) in animal experiments. An amplitude of the pulsatile component (APC) of iPPG waveform was assessed before and within 2.5 h after the NTG administration in saline (n = 13) or sumatriptan (n = 12) pretreatment anesthetized rats in conditions of a closed cranial window. In animals of both groups, NTG caused a steady decrease in blood pressure. In 7 rats of the saline group, NTG resulted in progressive increase in APC, whereas decrease in APC was observed in other 6 rats. In all animals in the sumatriptan group, NTG administration was accompanied exclusively by an increase in APC. Diametrically opposite changes in APC due to NTG indicate a dual effect of this drug on meningeal vasomotor activity. Sumatriptan acts as a synergist of the NTG vasodilating action. The results we obtained contribute to understanding the interaction of vasoactive drugs in the study of the headache pathophysiology and methods of its therapy.

## Introduction

Migraine is one of the forms of primary headache that affects about 14% of the global population according to recent data, which is a tangible medical and social problem^1,2^. Despite the obvious progress in understanding the neurobiology^3^ and treatment of this disease^4–6^, many issues of its pathogenesis remain controversial^7–9^, while the efficacy and safety of various therapies leave much to be desired^10,11^. The high prevalence, social significance, unmet need for therapy and the lack of a clear understanding of the mechanisms of migraine development contribute to the fact that this cephalgia is the object of intensive study.

Among the many experimental and clinical approaches to the study of the pathophysiology and treatment of migraine, the so-called “nitroglycerin model” occupies a prominent place^12^. It is known that in most patients with this pathology, nitroglycerin (NTG) with different routes of administration causes delayed migraine-like headache, often accompanied by prodromal symptoms and migraine-relative neurophysiological, vascular and metabolic reactions^13–17^, the appearance of which to a certain extent can be prevented or aborted with antimigraine drugs^18–20^. In animal experiments, systemic administration of NTG is accompanied by enhanced pain behavior and increased excitability and/or metabolic activity of neurons of the trigemino-thalamo-cortical pathway, which serves as the neuroanatomic basis of migraine. As a rule, these NTG-induced changes are sensitive to clinically effective antimigraine pharmacological agents^21–25^. The popularity of the NTG model in experimental cephalgology continues to be high, and the results obtained with its help regularly update ideas about the pathobiology of migraine^26–28^.

Against the background of an abundance of data on neuronal reactions induced by NTG, there is relatively insufficient information about how NTG affects intracranial (in particular, meningeal) vessels^12^, which looks like an undoubted omission, since migraine is considered to be a neuro-vascular pathology^3,29–31^. Indeed, the dural and pial arteries are positioned as one of the sources of pain^32,33^, and they are involved in the processes of spreading cortical depression^30,34^ and sterile meningeal inflammation^7,33,35^. Note that dilation of these vessels is considered as a reliable marker of trigeminal activation^36–38^. However, the uncertainty of points of view regarding the role of vasomotor reactions and the causal relationship between neuronal and vascular events in the pathogenesis of migraine^3,29^ dictates the need to obtain additional data, including using new methodological approaches.

We have recently shown that a multimodal approach using imaging photoplethysmography (iPPG) at green illumination synchronized with the electrocardiogram (ECG) can serve as a reliable tool for studying intracranial blood flow in rats under various experimental conditions^39–41^. In particular, we identified new optical markers of trigeminal afferents activation and determined their sensitivity^42^ to valproic acid and sumatriptan, two clinically effective drugs for preventive and abortive migraine treatment, respectively^43^. These features make it promising to use this approach to study various migraine-related interventions at the peripheral level of the trigeminovascular system. Taking into account our own positive experience of using iPPG + ECG system and the high informativeness of the NTG model, we set the following aims for this study: (i) to assess the effects of systemic administration of NTG on blood-flow parameters in meningeal vessels measured by iPPG in conditions of a closed cranial window; (ii) to evaluate the effect of 5-HT1B /1D receptor agonist sumatriptan on NTG-induced changes in the blood-flow parameters.

## Methods

### Animals

All experiments were carried out according to the ethical guidelines of the International Association for the Study of Pain, the Directive 2010/63/EU of the European Parliament and the Council on the protection of animals used for scientific purposes, and reported in compliance with the ARRIVE guidelines 2.0^44^. The study protocol was approved by the Institutional Animal Care and Use Committee of Pavlov First St. Petersburg State Medical University before carrying out the experiments. All efforts were made to minimize animal suffering and to reduce the number of experimental subjects necessary to produce reliable data. We used adult male Wistar rats (mean body weight 423 ± 82 g, n = 25) that were not previously subjected to experimentation and did not receive any drug. Animals were purchased from the State Breeding Farm ‘‘Rappolovo” (Saint Petersburg, Russia) and kept in groups (2–5 per cage) under standard laboratory conditions (12-h light/dark schedule) with food and water available ad libitum.

### Anesthesia and surgical preparation

Anesthesia and surgical preparations were performed as previously described^40,42^ with minor modifications. Briefly, the rats were anesthetized by intraperitoneal injection with a mixture of urethane (Sigma, St. Louis, MO, USA) and a-chloralose (Sigma, St. Louis, MO, USA) at an initial dose of 800/60 mg/kg. After achieving surgical anesthesia, each rat was placed on a thermostatically controlled heating pad, which provided a constant body temperature during whole experiment. The trachea was intubated for respiratory airflow and end-tidal carbon dioxide measurements. The right femoral artery and vein were cannulated for continuous arterial blood pressure (ABP) assessment and drug administration, respectively. The catheter was installed on anterior abdominal wall for intraperitoneal NTG administration. The animal’s head was mounted in a stereotaxic apparatus (Stoelting Co., Wood Dale, IL, USA). Closed cranial windows (CCWs) were formed by thinning the left and right parietal bones with a micro-drill to the state of a thin membrane, until the intracranial vessels became clearly visible through the remaining intact bone. During the drilling, tissues were cooled using topical application of cold saline.

All animals were paralyzed with pipecuronium bromide (“Arduan”, Gedeon Richter, Budapest, Hungary) at a dose of 1.0 mg/kg initially and maintained at 0.4 mg/kg, if necessary, and artificially ventilated with room air using a system SAR-830 (CWE, Inc., Ardmore, Pennsylvania, USA). During continuous video recording of meningeal vessels, the CCW surface was covered with mineral oil to minimize tissue dehydration and increase the transparency of the residual bone. In all experiments, the ECG was recorded using a digital electrocardiograph operating at a sampling frequency of 1 kHz (model KAP-01-Kardiotekhnika-EKG, Incart Ltd., Saint Petersburg, Russia). The contacts of the electrocardiograph were steel needles inserted into the muscle tissue of the rat limbs.

Continuous monitoring of ABP and end-tidal CO_2_ was carried out by the pressure sensor (MLT844, AD Instruments Inc., Colorado Springs, USA) and the carbon dioxide analyzer (Capstar-100, CWE, Inc., Ardmore PA, USA), respectively. These data were digitized at a sample frequency of 10 kHz (ADC-DAC Power1401-3, Cambridge Electronic Design, Cambridge, UK) and recorded on the personal computer using Spike2 version 8 software (Cambridge Electronic Design, Cambridge, UK). The adequacy of anesthesia was evaluated by the absence of the withdrawal reflex after paw pinch (before myorelaxation) or severe (> 20%) blood pressure fluctuations (after myorelaxation). If necessary, a supplemental dose of anesthetic mixture of urethane/α-chloralose was administered intravenously.

### Experimental protocol

All animals (n = 25) were randomly divided into two groups, one of which (n = 12, a treatment group) received an intravenous infusion of sumatriptan before NTG injection, while the second (n = 13, a control group) was instead pre-injected with saline in an equivalent volume (0.8 ml). Half of the animals in the treatment group were injected intravenously with sumatriptan (Tokyo Chemical Industry Co., Ltd., Japan) at a dose of 4 mg/kg dissolved in 0.8 ml of 0.9% NaCl, and the other half received a dose of 10 mg/kg of sumatriptan.

**Figure 1:**
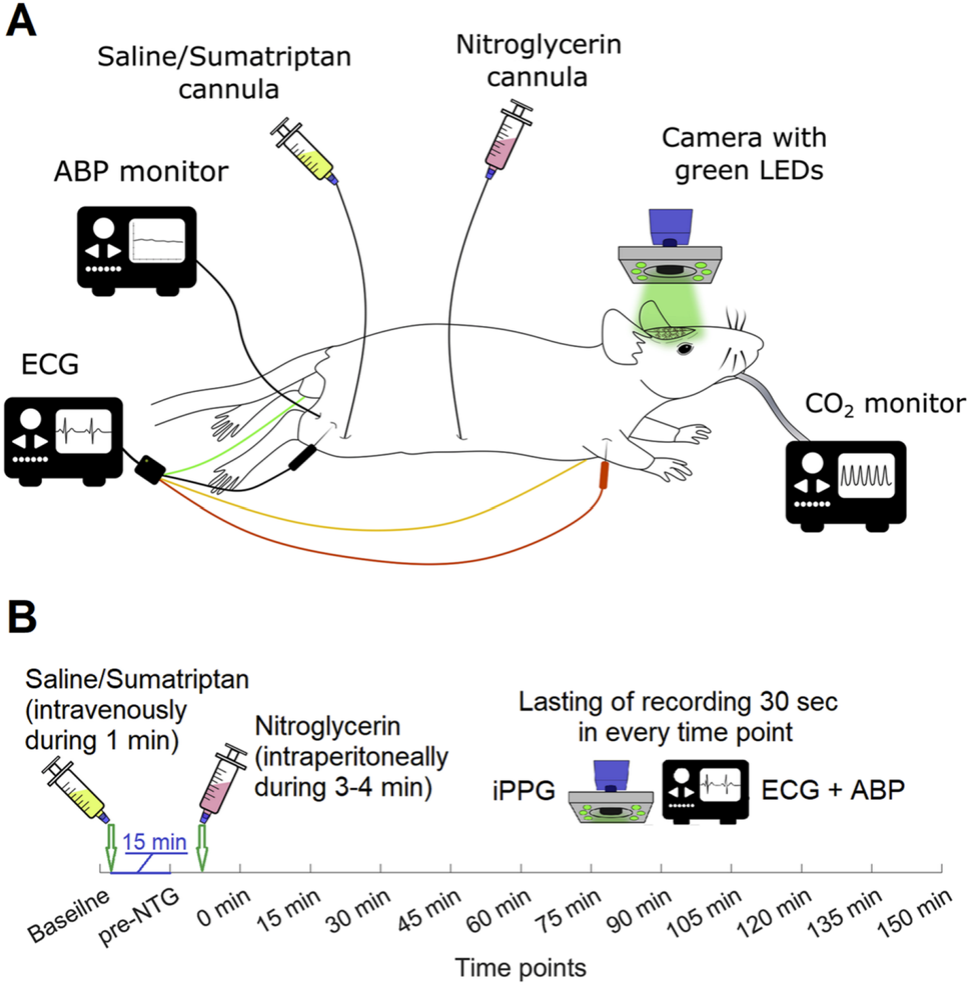
Experimental block diagram and measurement protocol. (**A**) Schematic of the experimental setup for monitoring the response of blood flow in intracranial vessels together with other physiological parameters to NTG administration. *ABP* arterial blood pressure, *ECG* electrocardiogram, *iPPG* imaging photoplethysmography. Nitroglycerin cannula was used for intraperitoneal administration of nitroglycerin. Cannulas in femoral vein and artery served for intravenous injection of sumatriptan or equivalent volume of saline, and for ABP monitoring, respectively. Steel needles were inserted into animal’s limbs for ECG recording by digital electrocardiograph. For monitoring of end-tidal CO_2_, a carbon dioxide analyzer was used. iPPG module was located above the rat’s brain to assess a reaction of the meningeal vessels through the closed cranial window. (**B**) Time-line of the experimental protocol. iPPG, ECG, and ABP were simultaneously and synchronously recorded during 30 s in every time point.

Each experiment included 13 consecutive 30-s video recordings of the state of the intracranial vessels through CCW with simultaneous recording of physiological parameters (ECG, ABP, heart rate, HR, and end-tidal CO_2_). Schematic of the experimental setup and the time-line of the measurement protocol are shown in Fig. 1. The first, baseline recording was made before injection of any drug. Next, either sumatriptan or saline was injected, and the second recording (a “pre-NTG” time point) was made in 15 min after the drug administration. Then the animals of both groups received NTG («Perlinganit», EVER Pharma Jena, GmbH; Germany) at a dose of 10 mg/kg via intraperitoneal catheter, and new recording of visible vessels was made immediately after injection (designated as an initial “post-NTG” time point corresponding to “0 min”) and after every 15 min within 150 min, along with the set of physiological parameters.

### Optical measuring system

Parameters of the blood flow in the intracranial vessels were measured using a custom-made iPPG system synchronized with a digital electrocardiograph. The design of the system is similar to that described in our previous paper^45^. Video recording of the intracranial vessels was performed by an iPPG module (see Fig. 1), which includes a digital monochrome CMOS camera (8-bit model GigE uEye UI-5220SE, Imaging Development Systems GmbH, Germany) and an illuminator with 8 light-emitting diodes (LEDs, model TDS-P003L4F07, TDS Lighting Co., China). To increase the uniformity of CCW illumination, LEDs (wavelength of 525 ± 25 nm, flux of 100 lm, and power 3 W per diode) were assembled around the camera lens (model M1214-MP2, Computar, Japan) with 25-mm focal length. To maintain the operating temperature of the LEDs, the illuminator chassis was made of aluminum. The camera lens and LEDs were covered by polarizing films with mutually orthogonal orientation thus reducing influence of specular reflections and motion artifacts^46^. The iPPG module has been aligned so that the normal to the CCW surface is as close to the optical axis of the camera lens as possible. This alignment allowed us to minimize the influence of the effect of ballistocardiographic artifacts^47^. The distance between the camera lens and the CCW was about 15 cm.

Video frames were recorded at 100 frames per second with a resolution of 752 × 480 pixels and were transmitted to a personal computer via Ethernet interface in PNG format. The ECG data in two leads were transmitted to a personal computer together with the synchro pulses of the camera via the USB 2.0 interface, thus providing synchronization of frames and ECG with an accuracy of about 1 ms. At the stage of data processing, the ECG signal served as a cardiac timing reference.

### Data processing

Processing of the frames and synchronously recorded ECG was carried out in the following steps.

1. Digital stabilization of tissue images was applied to minimize the influence of motion artifacts using an optical flow algorithm^48^ with floating time-window. Considering the multidirectional character of the motion of tissue sections caused by peristaltic, respiratory, and cardiac activity, we divided the whole frame into 16 × 16 pixels segments, and applied the stabilization algorithm to each of them independently. The duration of the floating time-window for stabilization was equal to the average cardiac cycle, determined by the positions of the R-peaks of the synchronously recorded ECG. All further calculations were performed with stabilized video data. To reduce the processing time, the operator manually selected the region for the analysis in the first frame of the baseline time-point, which included only the area of CCW with visible vessels. It was further divided into small regions of interest (ROI) sizing 11 × 11 pixels, which was about 250 × 250 µm^2^ in the CCW plane. At all subsequent time points, the selected region and ROIs remained the same and their position was always tied to the spatial features of the blood-vessels pattern.
2. The PPG waveform was calculated by averaging the pixel values in each ROI for every frame over a period of 30 s, which was the same for all time points. Usually, a PPG waveform consists of fast-varying at the heartbeat frequency alternating component (AC) and slowly varying component (DC)^45^. To compensate for uneven illumination, we calculated the AC/DC ratio in its absolute numerical values from the pixel-value waveform for each ROI, since both the AC and DC components are directly proportional to the incident light intensity^49^. The resulting PPG waveform was divided into segments equal to the corresponding cardiac cycles determined by the R-peaks of the synchronously recorded ECG. In each such segment for each ROI, the amplitude of the pulsations was calculated as the span of the PPG waveform.
3. The obtained set of the amplitudes of pulsations is first averaged over 30-s interval of iPPG recording, and then over all ROIs. It is worth noting that no significant differences in the amplitude of pulsations between different ROIs were observed. By this way, the amplitude of the pulsatile component (APC) was calculated for each time point during the experiment. This parameter was used to assess the hemodynamics of intracranial vessels. Similarly, the ABP was averaged over each respective 30-s interval of recording. HR was assessed using ECG data at the same time points.

All data processing was carried out offline on a computer running the Windows 10 operating system using custom-designed software implemented in the Matlab platform (Version R2021b, The MathWorks, Inc., MA, USA).

### Statistical analysis

For each of the 25 animals, 13 records of various physiological parameters (ABP, HR, and APC) with a duration of 30 s were analyzed. When comparing the dynamics of intracranial blood-flow among different groups of animals, the APC-parameter was normalized to that measured at the "pre-NTG" time point, taken as 100%; the dynamics of ABP and HR was compared in absolute values expressed in mmHg and bpm, respectively. Taking into account the small sample of animals in each of the experimental groups, nonparametric tests (Friedman, Kruskal–Wallis, Dunn's multiple comparisons, Wilcoxon signed-rank, and Mann–Whitney *U* tests) were used to assess the significance of changes in the studied hemodynamic parameters. All data were statistically analyzed using GraphPad Prism 8 (GraphPad Software Inc., San Diego, CA, USA). *P* < 0.05 was defined as statistically significant. For result description, the data were expressed as medians with interquartile ranges (Me [Q1–Q3]). Graphical presentation of the results was done using the Matlab software, the box plots were constructed in the style of Tukey.

### Ethics declarations

All experiments were carried out according to the ethical guidelines of the International Association for the Study of Pain, the Directive 2010/63/EU of the European Parliament and the Council on the protection of animals used for scientific purposes, and reported in compliance with the ARRIVE guidelines 2.0. The study protocol was approved by the Institutional Animal Care and Use Committee of Pavlov First St. Petersburg State Medical University before carrying out the experiments. All efforts were made to minimize animal suffering and to reduce the number of experimental subjects necessary to produce reliable data.

## Results

### Effect of NTG on systemic hemodynamics

#### NTG influence on the mean ABP

**Figure 2:**
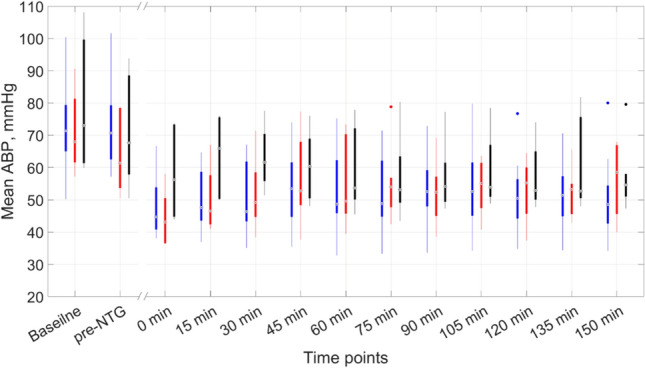
Dynamics of mean ABP changes after intraperitoneal administration of nitroglycerin. Time points in the X axis before the gap show moments of the ABP assessment in the baseline and at the 15th minute after intravenous infusion of either saline or sumatriptan (pre-NTG), whereas after the gap we show moments of the ABP measurements in minutes starting just after NTG administration. The blue boxes correspond to rats of the control group (saline pretreated), the red boxes are for rats of the sumatriptan-pretreated subgroup at a dose of 4 mg/kg, and black boxes are for rats pretreated with sumatriptan at 10 mg/kg. Significance of the differences is not shown, see the text to its assessment.

The baseline indices of the mean ABP were 71.4 [61.5–80.8] mmHg in the control group (n = 13) and 70.3 [61.4–88.3] mmHg in the sumatriptan-pretreated group (n = 12), and did not differ from each other (*P* = 0.94, *U* = 76, Mann Whitney test). In the control group, NTG injection resulted in a significant decrease in ABP (*P* < 0.0001, *Fr* = 65.4, Friedman test). After intraperitoneal administration of NTG, the mean ABP in each point of the time-course was significantly smaller than in the baseline and pre-NTG measures (all *P* < 0.05, Dunn's multiple comparisons test) as seen in Fig. 2 (blue boxes). Note that the saline infusion had no effect on blood pressure compared to baseline (*P* > 0.99, Dunn's multiple comparisons test), which is also confirmed by the pairwise comparison test (*P* = 0.64, Wilcoxon matched-pairs signed rank test).

In the sumatriptan-pretreated group (n = 12), NTG administration was also accompanied by a significant decrease in blood pressure (*P* < 0.0001, *Fr* = 52.7, Friedman test). Post-hoc analysis revealed that after NTG administration, the mean ABP measured at time points of 0, 15, 60–90, 120, and 135 min was significantly lower compared to the pre-NTG time point (all *P* < 0.05, Dunn's multiple comparisons test). However, the mean ABP was significantly lower at every point of the time course compared to the baseline (all *P* < 0.05), with the exception of the 30-min point having a "borderline" significance level (*P* = 0.056; Dunn's multiple comparisons test). Infusion of sumatriptan at any dose was accompanied by a noticeable transient decrease in blood pressure. At the same time, it is worth noting that the post-hoc test did not reveal statistical differences between the baseline and pre-NTG time point (*P* > 0.99, Dunn's multiple comparisons test), whereas significant differences occur in pairwise comparison (*P* = 0.002, Wilcoxon matched-pairs signed rank test).

When dividing the sumatriptan-pretreated group into subgroups according to the dosages of sumatriptan used, namely 4 mg/kg (n = 6, red boxes in Fig. 2) and 10 mg/kg (n = 6, black boxes in Fig. 2), it was found that sumatriptan administration at smaller dose resulted in a significant decrease in mean ABP compared to baseline (*P* = 0.03, Wilcoxon matched-pairs signed rank test), while sumatriptan at the dose of 10 mg/kg did not cause significant changes in mean ABP compared to baseline (*P* = 0.09, Wilcoxon matched-pairs signed rank test). Furthermore, there were no differences in mean ABP between the control group and any of the two sumatriptan-pretreated subgroups at every point of the time course from baseline to the 150-th minute (Kruskal–Wallis test with Dunn's multiple comparisons test), see Fig. 2.

### Influence of NTG on the heart rate

The HR assessment in the baseline was 427 [387–451] bpm in the control group (n = 13) and 417 [386–440] bpm in the sumatriptan-pretreated group (n = 12). No difference in HR between the groups were observed: *P* = 0.69, *U* = 70, Mann Whitney test. In the control group (saline-pretreated rats), the administration of NTG did not affect HR: *P* = 0.54, *Fr* = 10.9, Friedman test. Likewise, saline infusion did not affect HR compared to baseline (*P* > 0.99, Dunn's multiple comparisons test), which is confirmed by the pairwise comparison test: *P* = 0.5, Wilcoxon matched-pairs signed rank test.

According to the results of the Friedman test, the administration of NTG significantly affected HR in animals from the sumatriptan-pretreated group (*P* = 0.008, *Fr* = 27, Friedman test), whereas the post-hoc test did not reveal significant differences within the group compared to the pre-NTG time point (all *P* > 0.09, Dunn's multiple comparisons test). Sumatriptan infusion did not affect HR (*P* = 0.38, Dunn's multiple comparisons test), although significant differences occur in pairwise comparison (baseline vs pre-NTG): *P* = 0.003, Wilcoxon matched-pairs signed rank test.

Like for the mean ABP, the intergroup comparison did not reveal significant differences in HR between the control group and any of two sumatriptan-pretreated subgroups at any of the points of the time course from baseline to 150-th minute (Kruskal–Wallis test with Dunn's multiple comparisons test).

### Effect of NTG on the pulsation amplitude of intracranial vessels

**Figure 3:**
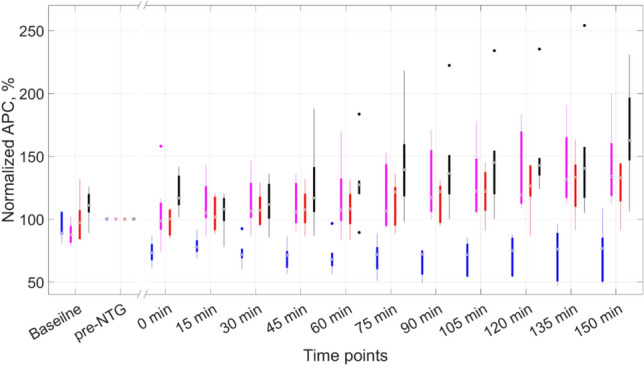
Dynamics of the APC index normalized to its value assessed at the pre-NTG time point in reaction to intraperitoneal administration of nitroglycerin. The time point designations on the X-axis are the same as in Fig. 2. Blue boxes correspond to rats of the control subgroup with the negative NTG-response, magenta boxes are for rats of the positive NTG subgroup, red boxes are for rats of the sumatriptan-pretreated subgroup at a dose of 4 mg/kg, and black boxes are for sumatriptan-pretreated rats at 10 mg/kg. Significance of the differences is not shown, see the text to its assessment.

In the control group (n = 13), the intraperitoneal administration of NTG lead to a divergent change in APC index: it significantly increased in 7 animals (*P* < 0.0001, *Fr* = 57.4, Friedman test) while a significant decrease in APC was observed in other 6 animals (*P* = 0.001, *Fr* = 32.7, Friedman test). Let us designate the former subgroup of the control group as a "positive NTG-responders", and the latter as a "negative NTG-responders". Time courses of changes in APC index in subgroups with positive and negative NTG response are shown in Fig. 3 by magenta and blue boxes, respectively. In the subgroup with positive NTG response (n = 7), the significance of differences in APC compared to the pre-NTG point was revealed at 120-th, 135-th and 150-th minutes after NTG administration (all *P* < 0.05, Dunn's multiple comparisons test), while with the pairwise comparison significant difference in APC was observed at each time point since 90-th minute (Wilcoxon matched-pairs signed rank test). In animals from the subgroup with negative NTG response (n = 6), the significance of differences in APC compared to the pre-NTG point was observed in the interval of 45–105 min and at 135-th minute after NTG administration: all *P* < 0.05, Dunn's multiple comparisons test. The pairwise comparison for this subgroup revealed that the differences are significant at each point of the time course from 0 to 135 min: all *P* < 0.05, Wilcoxon matched-pairs signed rank test (Fig, 3).

At each time-point of the time course after NTG administration we carried out the intergroup comparison of the normalized APC parameter in respect to the pre-NTG level using the Kruskal–Wallis test. No differences among the 4 subgroups were revealed in the baseline point (*H* = 7.3, *P* = 0.06), whereas after NTG administration, significant differences were found in every time point including zero: all *H* > 11.3, *P* < 0.01. These differences were clarified in the post-hoc Dunn's test, which revealed significant differences at most of time points between the subgroups of “negative NTG responders” and “positive NTG responders”, as well as between “negative NTG responders” and “sumatriptan-pretreated at 10 mg/kg” as can be seen in Table 1. Attention is drawn to the persistent significance of the differences between these pairs of subgroups determined at every point of the time course after NTG administration.

**Table 1:** Statistical significance of differences in APC changes relative to the pre-NTG point between the subgroups of animals at all points of the time course after NTG administration revealed by the post-hoc Dunn test.

Time-point, min	“Negative NTG responders” vs “positive NTG responders”, *P*-value	“Negative NTG responders” vs “sumatriptan at 10 mg/kg”, *P*-value
0	NS*	0.0007
15	0.008	NS†
30	0.01	0.03
45	0.02	0.005
60	0.03	0.006
75	0.03	0.002
90	0.01	0.003
105	0.02	0.003
120	0.02	0.003
135	0.01	0.01
150	0.02	0.002

*NS* no significant differences between the subgroups by the Dunn test.

*Clarification by the Mann Whitney test: *P* = 0.005, *U* = 2.

^†^Clarification by the Mann Whitney test: *P* = 0.02, *U* = 3.

To clarify some questionable results of the Dunn's test, the Mann Whitney test was used for pairwise comparison of unpaired data. This test revealed significant difference between subgroups listed in Table 1 in two time-points (see footnotes in Table 1) and in some points between the subgroups "negative NTG-response" and "sumatriptan-pretreated at 4 mg/kg" as shown in Table 2.

**Table 2:** Clarification of the borderline statistical significance of differences in APC between the subgroups by the Mann Whitney test.

Time-point, min	“Negative NTG responders” vs “sumatriptan at 4 mg/kg”, *P*-value	“Sumatriptan at 4 mg/kg” vs “sumatriptan at 10 mg/kg”, *P*-value
0	0.004, *U* = 1	NS
15	0.009, *U* = 2	NS
30	0.004, *U* = 1	NS
45	0.004, *U* = 1	NS
60	0.009, *U* = 2	NS
75	0.004, *U* = 1	NS
90	0.002, *U* = 0	NS
105	0.002, *U* = 0	NS
120	0.004, *U* = 1	NS
135	0.004, *U* = 1	NS
150	0.004, *U* = 1	0.04, *U* = 5

*NS* no significant differences between the subgroups by the Mann Whitney test.

It is worth noting that differences between the subgroups "negative NTG-responders" and "sumatriptan-pretreated at 4 mg/kg" were detected only in the Mann Whitney test, but not in the Dunn test for all the time points. At the 150-th minute, the significance of differences between the subgroups "sumatriptan-pretreated at 4 mg/kg" and "sumatriptan-pretreated at 10 mg/kg" was revealed by the Mann Whitney test, which was a somewhat expected result, given the significance of changes in APC compared to the pre-NTG time point within each of the subgroups. No significant changes were found between the subgroups "positive NTG-responders" and "sumatriptan-pretreated at 4 mg/kg", as well as between "positive NTG-responders" and "sumatriptan-pretreated at 10 mg/kg".

## Discussion

Our study revealed for the first time that NTG administration can lead to diametrically opposite changes in APC. Immediately after intraperitoneal administration of NTG, with a probability of about 0.5, there was a divergence in the APC evolution (either increase or decrease of this index was observed), which was progressing over time and stabilizing about 1.5–2 h after NTG injection. Since the systemic ABP was observed to decrease after NTG administration immediately and steadily in all cases, there is reason to believe that divergent APC trajectories reflect local intracranial vascular events and are hardly a consequence of changes in systemic hemodynamics. Accordingly, if we assume that APC is determined mainly by ABP, then in all cases we would register an exclusively either decrease or increase of this index.

The pulsatile component of the PPG waveform (assessed by APC) originates from the modulation of blood volume in the vessels with which this light interacts^50–52^. Green light used in our study did not penetrate deep into the brain cortex. However, the light interacts with a complex three-dimensional vascular network. Its intensity become modulated on HR as a result of blood volume oscillations either directly in visible arteries and arterioles^50^ or indirectly due to compression of the vascular network by transmural pressure^51^. Therefore, the magnitude of APC is related to summarized complex response of different vessels located at the measuring point to the pressure difference in systole and diastole. Depth of the vessels location that contribute to the modulation of light intensity can exceed the green-light penetration length into the tissue^52^. In the rat’s cranial cavity, when measured through CCW, the dural and pial small arteries and arterioles primarily contribute to the formation of PPG waveform. Since the change in blood volume associated with HR is regulated by vascular tone, the observed duality of the APC dynamics means multidirectional (both increasing and decreasing) change in the tone of the intracranial vessels. In a simplified sense, a decrease in the tone of the vascular wall should be accompanied by an increase in APC, whereas an increase in tone should lead to a decrease in APC^53^. The results of studies carried out by our group using both unconditional vasodilating agents, such as CO_2_^41^ or the carbonic anhydrase inhibitor dorzolamide^40^, and neurogenic dural vasodilation^42^ fully confirm such a performance.

NTG is also well-known vasodilator^54^. Consequently, its administration should be accompanied by vascular tone diminishing, but according to the results of the current study, it has a dual effect leading to both vasodilation and vasoconstriction. The dilation of meningeal vessels and / or local increase in blood flow due to NTG is quite an expected effect that has been time and again demonstrated in experiments on rodents by other authors with different methods of administration of NTG in different doses and for different periods of time after its administration. For example, NTG infusion (20 mcg/kg, i.v.) increased dural and pial artery diameter and caused an increase in local cortical cerebral blood flux within 8–12 min^55,56^; Laser Doppler scanning measurements revealed a gradual increase of meningeal blood flow reaching its maximum 2 h after NTG (10 mg/kg i.p.) injection^57^; pial arteriolar dilatation was observed almost immediately after NTG infusion (2 mg/kg/min, up to 10 mg/kg, i.v.) and reached its peak at 15 min and persisted for > 60 min^58^. On the contrary, the decrease in APC caused by NTG, which is probably associated with vasoconstriction and/or blood-flow diminishing contradicts well-established ideas about the pharmacodynamics of this drug and is an intriguing result. At the same time, it was previously shown that NTG can have a multidirectional effect on the tone of vessels of different caliber and localization, i.e., it has not only a classical dilating, but also a constricting effect. Thus, a case of paradoxical vasospastic reaction to intracoronary injection of NTG during coronary angiography is described^59^. It was shown in experiments on rats using two-photon laser scanning microscopy that arterioles react to NTG (10 mg/kg, intraperitoneal) in two opposing ways, i.e., by constriction in dura mater and dilation in the cortex. At the same time, the strong constrictive response of dural arterioles did not depend on the type of skull preparation (cranial window or thinned skull) and the type of anesthesia (кetamine/xylazine mixture or urethane). Moreover, both the lack of significant vasodilation in cortical arterioles under urethane anesthesia and weaker dilation of cortical vessels in the case of thinner skull were observed^60^. In other words, in animals under urethane anesthesia and with a closed cranial window (we have similar experimental conditions), only NTG-induced constriction of dural arterioles was de facto observed. The authors explain the opposite vasomotor activity of the dural and pial-cortical vessels by differences in their cellular microenvironment (mastocytes in the dura and astrocytes in the cortex), the degree of involvement in the formation of the blood–brain barrier, and the small diameter. These data are consistent with previous blood-flow assessment in rats using laser Doppler flowmetry, where it was observed that in the dura mater, NTG (10 mg/kg intravenously) resulted in a two-phase response, represented by an initial decrease in blood flow followed by a significant increase^61^, which indicates a transient constriction of dural arterioles. Chaliha et.al suggested that exogenous NO-donors cause dilation of relatively large arteries and compensatory contraction of microcirculatory vessels, which leads to tissue hypoxia, activation of cranial nociceptors and induction of pain^62^. This hypothesis is confirmed by the results of a previously conducted clinical study using 3 T magnetic resonance imaging in which was found that NTG did not influence the Blood-oxygen-level-dependent (BOLD) response^63^. The authors gave the following explanation: “the lack of effect of NTG on the BOLD response could be that NTG affects the macrovascular part of the vascular system and does not have an effect on the microvascular system that is involved in the BOLD mechanism.” Moreover, in this study it was shown that acetazolamide depressed the BOLD response probably by increasing cerebral blood flow, which is consistent with the results previously obtained in our group that the dorzolamide leads to an increase in APC^40^.

Comparing the results of the work^63^ with the data presented in the study^40^ we can draw interesting parallels: intravenous infusion of a carbonic anhydrase inhibitor causes rapid (within 5 min) depression of BOLD response in humans and an equally rapid (within 3 min) increase in APC in rats, whereas NTG administration does not change the BOLD response in humans and does not influence APC (if APC time-courses of the subgroups “positive NTG-response” and “negative NTG-response” are analyzed together). These findings open up new facets of the pharmacodynamics of these drugs and expand the understanding of their effects on cerebral hemodynamics. And finally, in a recent clinical study on healthy volunteers^64^, it was shown that dilatation of the middle cerebral artery due to NTG administration was accompanied by a significant decrease in compliance of the downstream cerebral vasculature, which, logically, should lead to a decrease in the amplitude of blood pulsations in intra-parenchymal arterioles.

Considering the above listed observations and taking into account the fact that iPPG waveform reflects pulsations summarized over different types of vessels, we conclude that two types of intracranial vascular response (constricting and dilating) to NTG were experimentally observed. Such an observation confirms the previously suggested assumption about differential region- and vessel-type-specific effects of NTG on cranial vessels^60^.

In our study, it was shown that intravenous infusion of the 5-HT1B /1D receptor agonist sumatriptan at doses of either 4 or 10 mg/kg 15 min before administration of NTG lead to exclusively progressive increase in APC in all 12 animals of the "sumatriptan-pretreated" group. This means that sumatriptan blocks the feature of NTG to induce a decrease in APC. Sumatriptan is often positioned as a vasoconstrictor^65^, which is not quite correct, because the actual vasoconstriction was demonstrated in experiments on isolated arteries^66–69^ while the "vascular" mechanism of action of triptans in migraine is rather 5-HT1B-mediated restoration of the tone of dilated cranial arteries, more precisely, triptans do prevent their dilation^69,70^. Considering that an increase in APC is due to a decrease in vascular tone, we may conclude that sumatriptan in both doses did not prevent the relaxation of the vascular walls caused by NTG, otherwise its administration would result in a decrease in APC, or at least, in stabilization at the baseline level. On the one hand, the result we obtained contradicts the accumulated experimental and clinical data. In particular, it was previously shown that (i) “in healthy male subjects sumatriptan prevents the effect of nitroglycerin-induced vasodilation in the middle cerebral artery”^71^; (ii) “sumatriptan reduced the NTG-induced headache and decreased temporal and radial artery diameters”^72^; (iii) “nitroglycerin-induced headache in individuals with migraine without aura is associated with blood flow velocity changes in the middle cerebral artery that are reversed by administration of an oral triptan”^73^. In experiments with rats, sumatriptan prevented NTG-induced increase in cortical blood flow, as assessed by laser Doppler flowmetry^74^.

On the other hand, there are observations that sumatriptan did not exhibit constricting properties in relation to the porcine isolated meningeal artery^75^. Moreover, even a dilating effect on various cranial arteries was shown ex vivo and in vivo for triptans that indicates the selectivity, dose dependence, and species specificity of their vascular action, for example, in relation to humans and rodents^68,76–78^. As for the above-mentioned "vascular" effects of the NTG and sumatriptan interaction, they also cannot be called unambiguous. So, using positron emission tomography and transcranial Doppler on 12 healthy volunteers, it was revealed that nitroglycerin increased global cerebral blood flow while flow velocities decreased, whereas sumatriptan did not have a significant effect on these values^79^. In a study performed on primates, according to single photon emission computed tomography, sumatriptan just amplified the increase in cerebral blood flow caused by NTG, allowing the authors to conclude that effective treatment with sumatriptan may therefore be compromised with simultaneous administration of nitroglycerin or NO donor drugs^80^. This kind of conclusion correlates with the results of a later study on arterioles isolated from human and bovine cerebral cortex in which the L-NNA (NO-synthase inhibitor) abolished the sumatriptan-induced dilation and shifted the dose–response of the constriction curve to the left^76^. Therefore, the opposite assumption seems to be true, saying that an excess of NO provoked by NTG can contribute to the development of triptan-induced vasodilation, especially with respect to small-caliber vessels. Indeed, sumatriptan and rizatriptan perfused through the lumen (intraluminal infusion) of isolated rat middle cerebral artery caused its dilation, at the same time, relaxant effect of sumatriptan was blocked by the 5-HT(1B/1D) receptor selective antagonist GR 55,562 and almost completely abolished by L-NOARG (NO synthase inhibitor), indicating nitric oxide involvement^77^. There is an explanation for these observations: it was shown in cultured human coronary artery endothelial cells that 5-HT-induced NO production is mediated by both of 5-HT1B and 5-HT2B receptor activation^81^; an endothelial expression of the 5-HT1B receptor was found in rat and human brain microvessels^82^. It is with the mechanism of endothelium-dependent vasodilation that the authors associate the fact that they have identified sumatriptan increases skin flap survival through activation of 5-hydroxytryptamine 1B/1D receptors and mediation of the nitric oxide system^83^. By the way, the involvement of the NO-signaling pathway in the mechanism of action of sumatriptan is noted by the authors of many modern preclinical studies to identify its new pharmacotherapeutic properties, in particular, cardioprotective^84^, antiallergic^85^, antipruritic^86^, and neuroprotective^87^ effects, as well as the study of its parenchymal toxicity^88^.

Therefore, in our study, sumatriptan acts as a synergist of the vasodilating action of NTG and prevents the manifestation of its "alternative" APC-inhibiting effect. This may be due, first, to the NO-mediated relaxing effect of sumatriptan on the walls of the meningeal microvessels and, second, to its constricting effect on the large intracranial arteries^89^, which will counteract the dilating effect of NTG on these vessels and thereby prevent compensatory constriction of the arterioles. A detailed study of the molecular mechanisms of the vascular reactions identified by us may become the subject of further research. The results we obtained speak in favor of the predominance of the neuronal mechanism of the antimigraine action of triptans over their vascular effects and contribute to understanding the nuances of the interaction of vasoactive drugs in the study of the headache pathophysiology and methods of anticephalgic therapy.

The limitations of this study include the absence of a control group for NTG itself, i.e., a separate cohort of animals that would receive a solvent instead of NTG. However, we purposely minimized the number of animals used in experiments, proceeding not only from the principles of humanism, but also from the idea that NTG is an indisputable trigger of migraine-like changes in various physiological parameters and controlling its effect is not mandatory in the light of a huge amount of experimental data.

## Data Availability

The data that support the findings of this study are available on request from the corresponding author (A.A.K.).
